# How abundant is a species at the limit of its distribution range? Crested porcupine *Hystrix cristata* and its northern population

**DOI:** 10.1002/ece3.10793

**Published:** 2024-01-26

**Authors:** Pablo Palencia, Stefania Zanet, Patricia Barroso, Rachele Vada, Francesco Benatti, Flavia Occhibove, Francesca Meriggi, Ezio Ferroglio

**Affiliations:** ^1^ Department of Veterinary Sciences University of Turin Torino Italy; ^2^ Ente Regionale per i Servizi all'Agricoltura e alle Foreste Milano Italy

**Keywords:** camera trap, conservation, distance sampling, population density, unmarked, wildlife

## Abstract

The crested porcupine (*Hystrix cristata*) is a rodent present in Africa and southern Europe (Italy exclusively). The Italian population is expanding from the centre to the north and south, but little is known about the species' abundance. Reliable population density estimates are important for monitoring trends in wildlife populations and for developing effective conservation and management strategies. In this context, we aimed to first report crested porcupine population density on the northern limit of its current distribution range using a non‐invasive approach. Specifically, we randomly placed 38 camera traps in an area of 242 km^2^ in north Italy (Lombardy region), and we applied camera trap distance sampling. We estimated a porcupine density of 0.49 ind·km^−2^ (±0.33, standard error). The results presented here are the first crested porcupine density estimate accounting for imperfect detection (i.e. species present but not detected). The abundance estimate reported here is fundamental for a better understanding of the species status in Europe and for implementing conservation and management plans.

## Graphical abstract

We first reported crested porcupine population density on the northern limit of its current distribution range using camera trapping. This is the first study to report crested population density in Europe, accounting for imperfect detection. The estimated density was 0.49 ind/km2.

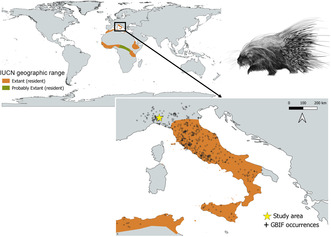

## 1 INTRODUCTION

The importance of better understanding the relationship between organisms and their environment has been identified as one of the five ‘major challenges’ in organismal biology (Schwenk et al., 2009). Measuring population density is important for monitoring trends in wildlife populations and for developing effective conservation and management strategies (Fryxell et al., 2014). In this respect, the crested porcupine (*Hystrix cristata*) represents a paradigmatic case of a species expanding its distribution range in Europe (Mori et al., 2013).

The crested porcupine is a rodent (family Hystricidae) distributed across central and northern Africa and southern Europe. Italy (both mainland and Sicilia) is the only country in Europe in which the crested porcupine is present, representing the northern distribution limit of the species. Since the 1970s, the crested porcupine has expanded its range into the northern regions of Italy, where it was historically absent (Mori et al., 2013). Briefly, the expansion of the distribution range in mainland Italy has been explained by three main factors: (i) the re‐expansion of woodlands (a consequence of agricultural abandonment), which is a paramount habitat for the species (Monetti et al., 2005); (ii) the climatic change, which broadly brings thermophilous species to reach higher latitudes (Winwood‐Smith et al., 2015); (iii) the legal protection, which probably reduced killing events for the species (Mori et al., 2013). The crested porcupine in Italy is strictly protected under the National Law 157/1992, whereas at the European level, it is included within the Annex II of the Bern Convention (1979) and the Annex IV of the ‘Habitat’ Directive 1992/43/EEC. The crested porcupine is also generating some human–wildlife conflict, especially related to vegetation damage (Laurenzi et al., 2016). In central Italy (Provinces of Florence and Grosseto), it has been reported that the damage caused by crested porcupines represents less than 5% of the overall damage caused by wildlife in agricultural crops, and less than 6% of the trees in a forest was debarked (Laurenzi et al., 2016).

**FIGURE 1:**
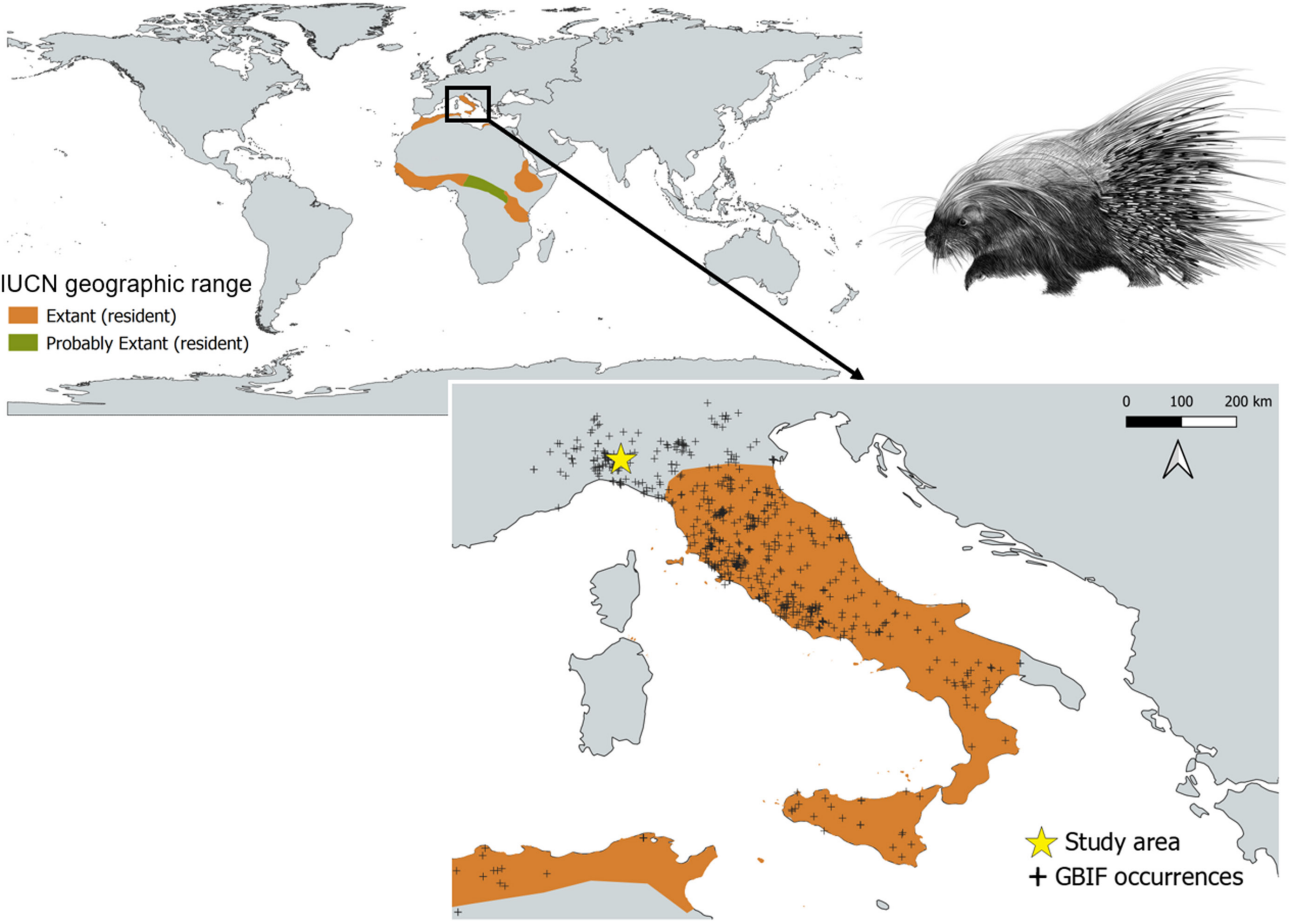
Crested porcupine (*Hystrix cristata*) distribution range according to the IUCN (International Union for Conservation of Nature, https://www.iucnredlist.org/species/10746/22232484#geographic‐range, accessed on 20 May 2023). Orange represents areas in which the presence of the species has been confirmed, while green represents areas with a probable presence but not confirmed. The bottom panel zooms in on the crested porcupine distribution in Europe. Black crosses indicate the species records reported to GBIF (Global Biodiversity Information Facility, https://www.gbif.org/species/5219888, accessed on 20 May, 2023), while the yellow star is the study area location. For interpretation of the references to colour in this figure legend, the reader is referred to the web version of this article.

In Italy, the species is homogeneously distributed in the central regions (Figure 1), while in both northern and southern areas it is present with a much more fragmented distribution (Mori et al., 2021). Despite a wide range of environmental parameters, such as land use, feeding habitats, occupancy etc., have been described (Mori et al., 2013, 2017, 2021, 2022), little is known about the population density (i.e. the number of individuals within an area) and demographic trends. The lack of data on regional and national abundance and densities in Italy makes challenging porcupine population management (https://www.gbif.org/species/5219888). To the best of our knowledge, only one study estimated porcupine abundance in the core area of the distribution range (Franchini et al., 2022). This study used presence‐only data to estimate abundance but did not account for imperfect detection—that is species not detected despite being present—(Dorazio, 2014; Franchini et al., 2022). The absence of porcupine population density data could be explained by the practical implications of monitoring an elusive species occurring at very low densities and without natural marks that allow the individual recognition of the animals.

In this context, the use of remotely activated cameras (camera traps) has been described as a non‐invasive, reliable methodology that overcame most of the limitations of previous methodologies (Rovero & Zimmermann, 2016; Wearn & Glover‐Kapfer, 2019). Camera traps are particularly useful for monitoring elusive species and can gather large quantities of data more quickly than other traditional survey methods (Burton et al., 2015). Focusing on the estimation of population density, methodologies to estimate population density without the need to individually identify animals (unmarked methods) have been described (Gilbert et al., 2021). Among them, camera trap distance sampling (CTDS) has been specially recommended when monitoring low‐abundant populations (Palencia et al., 2021).

Here, we aim to first report crested porcupine population density on the northern limit of its current distribution range. This is an important contribution to the assessment of the crested porcupine population status, with the final objective of informing management and conservation strategies.

## 2 MATERIALS AND METHODS

### 2.1 Study area

The study was carried out in the Lombardy region, Pavia province (north Italy, Lat: 45,53° N, Long: 9,10° E), within a fragmented mountainous area of approximately 242 km^2^ (Figure 1). The study area is dominated by open grassland areas and *Quercus*, *Pinus* and *Castanea* forests, with medium‐to‐high habitat suitability for crested porcupine, according to a recent study (Torretta et al., 2021). Altitudes ranged from 236 to 1684 m.a.s.l., and the climate is continental‐temperate.

### 2.2 Data collection

CTDS was considered to estimate porcupine population density (Howe et al., 2017). From November 2022 to February 2023 (83 days), 38 camera traps (Browning Strike Force HD X Pro‐model BTC‐5HDPX) were randomly deployed, covering the whole study area and habitats, without using attractants. If cameras could not be placed in the exact predetermined random location due to land access, terrain slope or other reasons, they were placed at the nearest suitable point within the same habitat and without targeting placement to increase or decrease detection probability. The mean distance between cameras was 1178.50 m (min: 727.68, max: 1790.31 m). Cameras were deployed heading towards the north, 50 cm above ground, with the sensor angled parallel to the slope. Cameras were set to be operative all day to record a burst of eight consecutive pictures at each activation, with the minimum time lapse between consecutive activations. Nocturnal pictures were illuminated with an infrared flash (low glow). Cameras were checked once a month to check the status of the batteries and memory cards. Animal‐to‐camera detection distances are required to apply CTDS (Howe et al., 2017). We applied a photogrammetry approach to estimate the location of the animals in the field of detection and lastly estimate animal‐to‐camera distances (Palencia et al., 2023; Wearn et al., 2022). Briefly, the photogrammetry approach describes (i) the relationship between the size of the calibration object in the image (in pixels) and its actual size and distance from the camera; and (ii) the relationship between the X‐Y pixel position in the image and the angular distance from the camera's principal axis. To apply the photogrammetry procedure, it is necessary to calibrate each camera deployment and the camera model. The camera model (here Browning Strike Force HD X Pro‐model BTC‐5HDPX) was calibrated once by taking images of the calibration pole (here a 1‐m‐length pole with marks at 20 cm intervals) at known distances from the camera (maximum 15 m) and from the centre to the laterals of the field of view. On the field, each camera deployment was calibrated by recording 15 pictures of the calibration pole across the field of view and spaces 1–2 m apart. We finally digitised the images to extract the pixel position of the porcupine in the open‐source tool *AnimalTracker*. Pixel positions were later transformed to animal‐to‐camera distances and angles using the CTtracking package in R.

### 2.3 Camera trap distance sampling analysis

CTDS estimates density from the following equation (Howe et al., 2017):

(1) $$\hat{D}=\frac{\sum_{k=1}^{K}n_{k}}{\pi w^{2}\;\sum_{k=1}^{K}e_{k}{\hat{P}}_{k}}\cdot \frac{1}{\hat{A}}$$

where *n*_*k*_ is the number of observations of animals in the population of interest at point *k*, *K* is the set of sampling points, *w* is the truncation distance beyond which any recorded animal is discarded, *e*_*k*_ = ƟT_*k*_/2π*t* is the sampling effort at point *k*, *Ɵ* is the angle of view of the camera, *T*_*k*_ is the sampling period and the predetermined set of snapshot moments *t* units apart. The snapshot moments are the opportunities to obtain an image of an animal. ${\hat{P}}_{k}$ is the estimated probability of obtaining an image of an animal that is within the detection zone at a snapshot moment. Finally, *Ȃ* is the activity level of the population (i.e. the proportion of time that the population spent in movement). All these parameters were calculated for the study population as described below.

#### 2.3.1 Snapshot moment

The density estimates obtained through CTDS are highly dependent on the values for the snapshot interval (McKaughan et al., 2023), which lastly determines survey effort (e_*k*_ Equation 1). In this respect, it has been demonstrated that the settings provided in the camera trap user manuals are not reliable (McKaughan et al., 2023; Palencia, Vicente, et al., 2022). We estimated the snapshot interval by dividing the time gap between consecutive pictures in which the porcupine was recorded by the number of pictures. Those pictures in which porcupines were recorded were exclusively considered to estimate the snapshot interval.

#### 2.3.2 Detection probability and model selection

To estimate detection probability ($\hat{P}$, eqn1), we fitted detection functions to model the decrease in detection probability as the distance between porcupines and cameras increases. The individual was considered as the unit of observation. When more than one porcupine was recorded on a picture, we recorded the detection distance and angle of each individual (Thomas et al., 2010). Treating individuals as the unit of observation minimised bias and performed as well or better than analysing data from groups (Buckland et al., 2010). When fitting distance sampling models, data were right‐truncated at 9 m due to the low probability of detection (Buckland et al., 2001) and left‐truncated at 2 m due to the paucity of observation closer to the camera, likely explained by animals passing beneath the PIR sensor. We fitted models of the detection function with the half‐normal key function with 0, 1 or 2 Hermite polynomial adjustment terms, the hazard rate key function with 0 or 1 cosine adjustments and the uniform key function with 1, 2 or 3 cosine adjustments. The best model was selected using QIAC and following a two‐step method (i.e. model selection within key functions (step 1) and model selection among key functions (step 2), Howe et al., 2019). Measures of uncertainty were derived from 999 bootstraps resampled, with replacement, across camera locations; and accounting for uncertainty in encounter rate, probability of detection and availability for detection.

#### 2.3.3 Availability for detection

The proportion of time active ($\hat{A}$, Equation 1) represents the availability of the population for detection. Camera traps record animals only when they move outside refuges, and resting animals are never detected (Rowcliffe et al., 2014). We estimated $\hat{A}$ by fitting a circular kernel model to radian time data using the R package ‘activity’ (Rowcliffe et al., 2014). The activity level and its standard error were lastly considered as a multiplier when estimating population density (Buckland et al., 2001).

## 3 RESULTS

We recorded 166,719 pictures, of which 232 corresponded to crested porcupines (on 11 of them, two individuals were recorded) on a total effort of 2744 trap nights. Crested porcupine was only detected in six out of 38 cameras, and no porcupine reacted to the camera. No camera was stolen. We only discarded two sampling points due to the irregular terrain in which the cameras were deployed, which led to biased animal locations derived from the photogrammetry method. The average snapshot estimated was 0.73 s (Equation 1).

**FIGURE 2:**
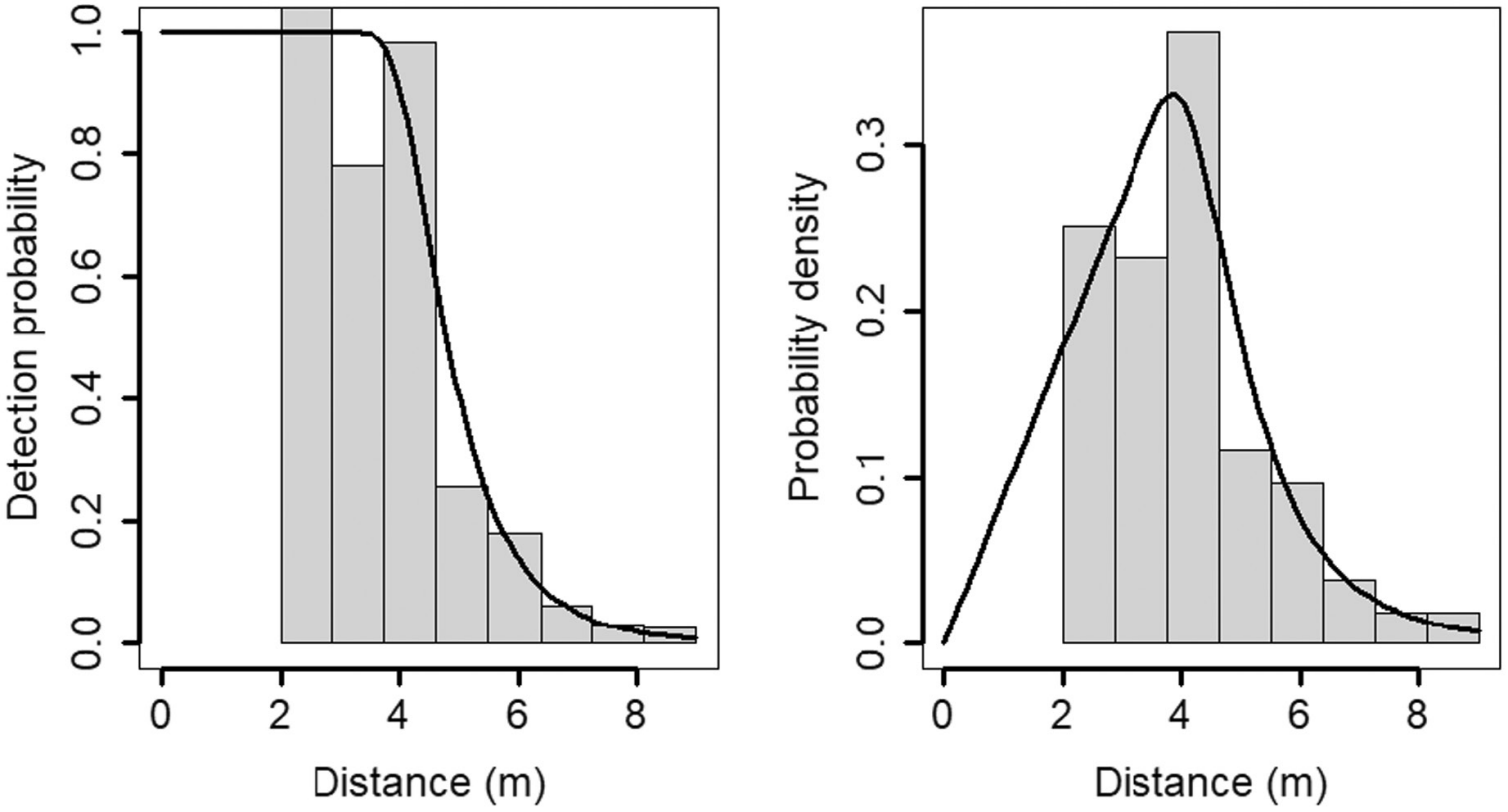
Detection probability graph (left panel) and probability density graph (right panel) as a function of distance for the best model selected (hazard rate without adjustment terms).

On the visual inspection of detection angles, we observed that detection probability decreased as the detection angle increased (Appendix S1). In consequence, we truncated the observations beyond 0.1 radians, as the distribution of detections departed from a uniform distribution (Howe et al., 2017). The *Ɵ* (Equation 1) parameter was set to 0.2 radians as we assumed a symmetric detection zone. The estimated activity level was 0.22 (±0.04), indicating that the population spends less than 6 h per day being active. After applying the model selection procedure, the best model fitted was the hazard rate without adjustment terms (Table 1, Figure 2, Appendix S1). We did not find overdispersion in the best model ($\hat{c}$ = 0.66, Appendix S1). The crested porcupine density estimated was 0.49 ind·km^−2^ (±0.33, standard error). The vast majority (80.85%) of the variance of the density was attributable to the variation in encounter rate between cameras; 12.72% was attributable to the availability of detection; and only 6.42% to detection probability.

**TABLE 1:** Camera trap distance sampling parameters estimated for the best model selected (hazard rate without adjustment terms).

Parameter	Parameter description	Estimate
*n*	Number of detections	59
*K*	Number of sampling points	36
*w*	Truncation distance (m)	9
*T*	Sampling effort (s)	326,426,301
$\hat{t}$	Snapshot (s)	0.73
*Ɵ*	Angle (rad)	0.2
*e*	Sampling effort	10,390,471.87
$\hat{P}$	Probability of detection	0.28
$\hat{A}$	Availability of detection	0.22
$\hat{D}$	Population density (ind·km^−2^)	0.49

## 4 DISCUSSION

We reported a population density of 0.49 porcupines·km^−2^ near the northern limit of its distribution range. We showed that CTDS might provide a practical method to facilitate monitoring the population density of crested porcupine, an elusive species, with nocturnal activity patterns occurring at low density.

Crested porcupine is expanding its distribution range and has recently colonised southern and northern Italy (Mori et al., 2013, 2021). Recent studies have also suggested that most of mainland Italy has high habitat suitability for the species, and thus an expansion in its distribution range is expected in the coming years (Mori et al., 2013, 2021). In this respect, since porcupine is considered a potentially problematic species due to the damage to croplands and riverbanks (Laurenzi et al., 2016), reliable data on its abundance is a priority aspect to implement conservation and management actions. In the province of Pavia, where the study was carried out, damage by crested porcupines during 2022 was valued at 252,93 €, representing 100% of the overall damage caused by wildlife. Population trend data is lacking (Amori & De Smet, 2016). To the best of our knowledge, no population densities on the border of the distribution range have been reported; only one study estimated porcupine density at the centre of the distribution area based on presence‐only data (Franchini et al., 2022). Franchini et al. (2022) reported population densities of 0.44 ind·km^−2^ at a regional scale (17,111 km^2^) while we worked at a local scale (242 km^2^), for which the results are not directly comparable. Additionally, they also suggested that lower densities (ca. 0.10 ind·km^−2^) could be more closely related to the true number of individuals. The slight difference between our density and the one previously reported could then be explained by (i) the different scales of both studies, (ii) the different methodologies applied (while Franchini et al. (2022) used presence‐only data, here we applied CTDS accounting for imperfect detection) and (iii) by the high habitat suitability for crested porcupine in our study area (Torretta et al., 2021), where higher densities are expected. These results are valuable for delineating adequate conservation and management plans. First, the distribution range should be updated as the species has expanded to the north (Figure 1). Second, the area surveyed here is characterised by open grasslands combined with deciduous and pine forests, but just 5 km to the north is located the Po Plain, an area between the Apennines and the Alps dominated by agricultural crops including corn, cereal, alfalfa and riparian habitats. The Po Plain is a key area for the species expansion towards the northernmost regions of Italy, mainly by the natural and semi‐natural vegetation cover along rivers, for example, the Po and Ticino rivers (Torretta et al., 2021). The camera trapping‐based methodology applied here could be considered the reference method to monitor crested porcupine density.

Distance sampling with camera traps has been widely applied to estimate mammal population density (Bessone et al., 2020; Cappelle et al., 2021; Mason et al., 2022; McKaughan et al., 2023). In this study, we reinforced the utility of CTDS to monitor low‐abundant populations (Palencia et al., 2021). In this respect, we would like to discuss some peculiarities found in our data. First, animal reaction to cameras is a common situation when monitoring wildlife and can lead to bias in the population estimates when applying CTDS (Delisle et al., 2023; Houa et al., 2022). While more than 50% of reactive animals have been observed in some populations (Houa et al., 2022), we did not find any reaction from porcupines to the cameras. This makes camera traps in general, and CTDS in particular, a reference method to monitor porcupine abundance. Second, we observed a marked decrease in the number of detections at higher angles (Appendix S1). While in early applications of CTDS the angle of the cameras provided by the manufacturer was considered as a reference, our results and other recent studies reinforce the need to estimate not only detection distance but also detection angle for the individuals recorded (Mason et al., 2022; McKaughan et al., 2023; Palencia, Vicente, et al., 2022). Third, similarly to the angle estimation, more recent CTDS studies have also focused on the sensitivity of density estimated to the snapshot interval definition (McKaughan et al., 2023). Once again, the deviation between the camera's handbook and the camera's performance on the field has been demonstrated (McKaughan et al., 2023). While McKaughan et al. (2023) estimated a snapshot interval of 2.11 s for the same camera model we used (Browning Strike Force HD X Pro‐model BTC‐5HDPX), we estimated an average interval of 0.73 s. We could explain this divergence due to McKaughan et al. (2023) using human trials and accounting for multiple activations between consecutive bursts. However, we based our estimation of snapshot interval exclusively on porcupine encounters because of (i) the inter‐species and inter‐device variability in camera performance previously demonstrated (Palencia, Vicente, et al., 2022) and (ii) the low number of porcupine encounters in which more than one burst was recorded. On average, we recorded 6.14 pictures per porcupine encounter. Thus, considering that eight pictures were automatically recorded in each burst, the time gap within a burst had more influence than the time gap between bursts in the definition of the snapshot for our target species. This could emphasise the need to estimate species‐specific snapshot intervals and not only one survey‐specific snapshot interval. Accordingly, our fourth point to be discussed is the overdispersion in the data. When applying CTDS, overdispersion is expected (and usually found) because we measured distances to the same animal several times. Analytical procedures have been described to deal with overdispersed data (Howe et al., 2019). However, we did not find overdispersion in our data (Appendix S1). As described above, we could explain the lack of overdispersion in our data due to the low number of distances measured to the same individual (6 on average), while more than 20–30 distances are frequently measured when applying CTDS (Palencia et al., 2021). Finally, in line with previous CTDS studies, the low precision in density was explained by the high variability in the encounter rate among cameras (Cappelle et al., 2021; Howe et al., 2017). For future studies, increasing the number of sampling points would be desirable to increase the precision. Nevertheless, low precision is not a CTDS‐specific issue with regard to population density estimation; in fact, most other methodologies employed to estimate population density without individual identification might show low precision (Palencia, Barroso, et al., 2022).

In conclusion, we have reported crested porcupine density at the limit of its distribution range, and we have discussed the utility of CTDS as a cost‐effective method. The elusive behaviour, nocturnal activity patterns and absence of natural marks to individually recognise animals limit the applicability of traditional survey methods on porcupines. The underlying advantages of camera trapping in general, and CTDS in particular, broaden and strengthen the applicability of this methodology to estimate reliable crested porcupine abundance, ultimately to be used to develop management and conservation strategies.

## AUTHOR CONTRIBUTIONS

**Pablo Palencia:** Conceptualization (lead); data curation (equal); formal analysis (lead); investigation (lead); methodology (lead); writing – original draft (lead). **Stefania Zanet:** Data curation (equal); funding acquisition (lead); investigation (supporting); project administration (lead); supervision (lead); writing – review and editing (equal). **Patricia Barroso:** Data curation (equal); writing – review and editing (equal). **Rachele Vada:** Data curation (equal); writing – review and editing (equal). **Francesco Benatti:** Data curation (equal); writing – review and editing (equal). **Flavia Occhibove:** Data curation (equal); writing – review and editing (equal). **Francesca Meriggi:** Data curation (equal); writing – review and editing (equal). **Ezio Ferroglio:** Funding acquisition (lead); investigation (supporting); project administration (lead); supervision (lead); writing – review and editing (equal).

## CONFLICT OF INTEREST STATEMENT

The authors declare no conflicts of interest.

## Supporting information


Appendix S1.

Appendix S2.

## Data Availability

Data are available in Appendix S2.
