# Supplementary material for: How abundant is a species at the limit of its distribution range? Crested porcupine Hystrix cristata and its northern population
Source: Ecol Evol. 2024 Jan 26;14(1):e10793. doi: 10.1002/ece3.10793 (PMC10811963; doi:10.1002/ece3.10793)
Supplement: Supplementary file 1 — Appendix S1. [file ECE3-14-e10793-s002.docx]

**How abundant is a species at the limit of its distribution range? Crested Porcupine *Hystrix cristata* and its northern population**

Pablo Palencia^1*^, Stefania Zanet^1^, Patricia Barroso^1^, Rachele Vada^1^, Francesco Benatti^1^ Flavia Occhibove^1^ Francesca Meriggi^2^, and Ezio Ferroglio^1^

1. Università Degli Studi di Torino, Dipartimento di Scienze Veterinarie, Largo Paolo Braccini, 2, 10095 Grugliasco Torino, Italy

2- Ente Regionale per i Servizi all’Agricoltura e alle Foreste, Via Pola 12, 20124 Milano, Italy

*Corresponding author: Università Degli Studi di Torino, Dipartamiento di Scienze Veterinarie, Largo Paolo Braccini, 2, 10095 Grugliasco Torino, Italy. E-mail. [palencia.pablo.m@gmail.com](mailto:palencia.pablo.m@gmail.com)

**Appendix S1:** camera trap distance sampling analysis details

**Distribution of detection angles**


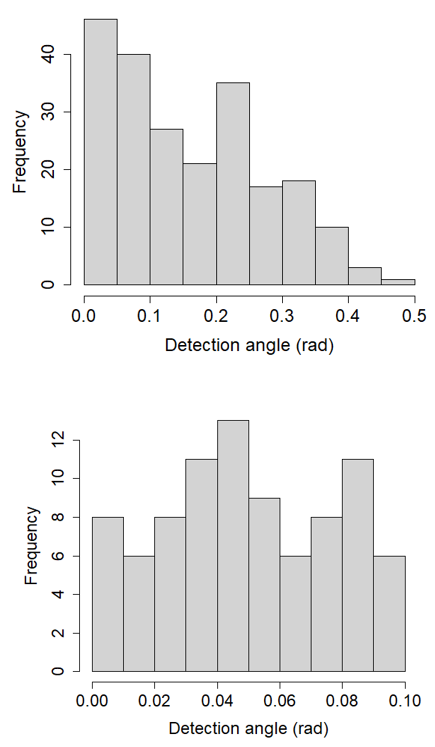


**Figure 1**: Detection angles in radians of porcupine detections. The upper panel represents all the detections, while the bottom panel represents those filtered by < 0.1 radians.

**Model selection procedures**

*Two-step procedure described by Howe et al. 2019*

Step-1:

**Table 1**: Point transect distance sampling model fitted to porcupine data. The lowest QAIC values for model selection within key functions (step-1 according to Howe et al. 2019) are in bold.

| **Key** | **Adjustment terms** | **QAIC** |
| --- | --- | --- |
| Uniform | 1 | **107.65** |
| Uniform | 2 | 112.06 |
| Uniform | 3 | 110.02 |
| Half-normal | 0 | **176.19** |
| Half-normal | 1 | 177.35 |
| Hazard rate | 0 | **232.26** |
| Hazard rate | 1 | 234.26 |

Step-2:

**Table 2**: Overdispersion ($\hat{c}$) results for distance sampling models selected in step-1.

| **Key** | **Adjustment terms** | $\hat{\boldsymbol{c}}$ |
| --- | --- | --- |
| Uniform | 1 | 1.94 |
| Half-normal | 0 | 1.06 |
| Hazard rate | 0 | **0.66** |

As shown in Table 2, the overdispersion parameter estimated for the three models selected in step-1 is close to one. In this respect, $\hat{c}$ = 1 indicates no overdispersion in the data, and as $\hat{c}$ increase, the higher the level of overdispersion.

In this respect, as the data are not overdispersed, the procedure described by Buckland et al. 2001 should be considered to select the most parsimonious model.

*Traditional procedure described by Buckland et al. 2001*

**Table 3**: Point transect distance sampling model fitted to porcupine data. The lowest AIC value for model selection is in bold.

| **Key** | **Adjustment terms** | **AIC** |
| --- | --- | --- |
| Uniform | 1 | 197.52 |
| Uniform | 2 | 204.07 |
| Uniform | 3 | 198.46 |
| Half-normal | 0 | 192.30 |
| Half-normal | 1 | 193.38 |
| Hazard rate | 0 | **191.55** |
| Hazard rate | 1 | 193.54 |

Interestingly, the hazard rate model without adjustment terms was selected as the best model by both procedures.

**Bootstrapping results**


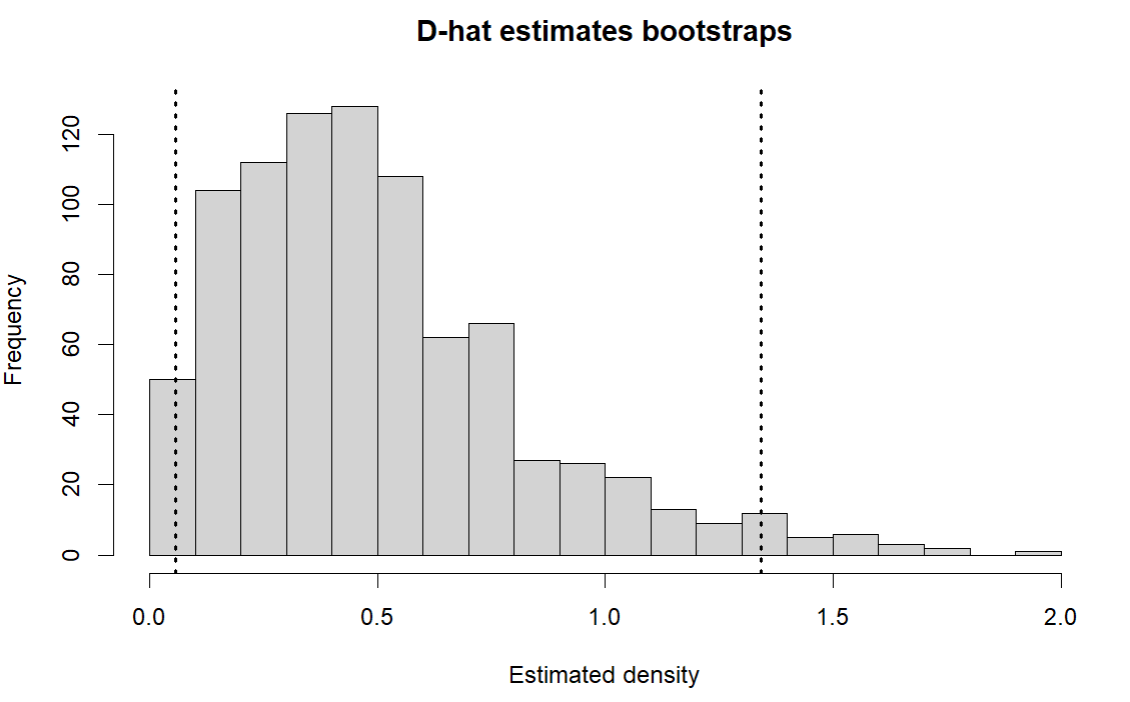


**Figure 2**: Distribution of density estimates from 999 bootstrap estimates. Vertical dotted lines represent the 95% confidence interval limits.

**References**

Buckland, S. T., Anderson, D. R., Burnham, K. P., Laake, J. L., Borchers, D. L., & Thomas, L. (2001). *Introduction to Distance Sampling: Estimating Abundance of Biological Populations*.

Howe, E. J., Buckland, S. T., Després‐Einspenner, M., & Kühl, H. S. (2019). Model selection with overdispersed distance sampling data. *Methods in Ecology and Evolution*, *10*(1), 38–47. https://doi.org/10.1111/2041-210X.13082
